# Systematic review: fluid biomarkers and machine learning methods to improve the diagnosis from mild cognitive impairment to Alzheimer’s disease

**DOI:** 10.1186/s13195-023-01304-8

**Published:** 2023-10-14

**Authors:** Kevin Blanco, Stefanny Salcidua, Paulina Orellana, Tania Sauma-Pérez, Tomás León, Lorena Cecilia López Steinmetz, Agustín Ibañez, Claudia Duran-Aniotz, Rolando de la Cruz

**Affiliations:** 1 Center for Social and Cognitive Neuroscience (CSCN), School of Psychology, Universidad Adolfo Ibanez, Diagonal Las Torres 2640, Peñalolén, Santiago, Chile; 2 Latin American Institute for Brain Health (BrainLat), Universidad Adolfo Ibáñez, Santiago, Chile; 3 Faculty of Engineering and Sciences, Universidad Adolfo Ibáñez, Diagonal Las Torres 2700, Building D, Peñalolén, Santiago, Chile; 4 Global Brain Health Institute, Trinity College, Dublin, Ireland; 5 Memory and Neuropsychiatric Center (CMYN) Neurology Department, Hospital del Salvador and Faculty of Medicine, University of Chile, Santiago, Chile; 6 Technische Universität Berlin, Berlin, Deutschland; 7 Instituto de Investigaciones Psicológicas (IIPsi), Universidad Nacional de Córdoba (UNC) y Consejo Nacional de Investigaciones Científicas y Técnicas (CONICET), Córdoba, Argentina; 8 Global Brain Health Institute, University of California San Francisco (UCSF), San Francisco, CA USA; 9 Cognitive Neuroscience Center (CNC), Universidad de San Andrés, & National Scientific and Technical Research Council (CONICET), Buenos Aires, Argentina; 10 Data Observatory Foundation, ANID Technology Center No. DO210001, Santiago, Chile

**Keywords:** Mild cognitive impairment, Alzheimer’s disease, Fluid biomarker, Machine learning, Artificial intelligence

## Abstract

Mild cognitive impairment (MCI) is often considered an early stage of dementia, with estimated rates of progression to dementia up to 80–90% after approximately 6 years from the initial diagnosis. Diagnosis of cognitive impairment in dementia is typically based on clinical evaluation, neuropsychological assessments, cerebrospinal fluid (CSF) biomarkers, and neuroimaging. The main goal of diagnosing MCI is to determine its cause, particularly whether it is due to Alzheimer’s disease (AD). However, only a limited percentage of the population has access to etiological confirmation, which has led to the emergence of peripheral fluid biomarkers as a diagnostic tool for dementias, including MCI due to AD. Recent advances in biofluid assays have enabled the use of sophisticated statistical models and multimodal machine learning (ML) algorithms for the diagnosis of MCI based on fluid biomarkers from CSF, peripheral blood, and saliva, among others. This approach has shown promise for identifying specific causes of MCI, including AD. After a PRISMA analysis, 29 articles revealed a trend towards using multimodal algorithms that incorporate additional biomarkers such as neuroimaging, neuropsychological tests, and genetic information. Particularly, neuroimaging is commonly used in conjunction with fluid biomarkers for both cross-sectional and longitudinal studies. Our systematic review suggests that cost-effective longitudinal multimodal monitoring data, representative of diverse cultural populations and utilizing white-box ML algorithms, could be a valuable contribution to the development of diagnostic models for AD due to MCI. Clinical assessment and biomarkers, together with ML techniques, could prove pivotal in improving diagnostic tools for MCI due to AD.

## Introduction

Mild cognitive impairment (MCI) is defined as a heterogeneous clinical syndrome including cognitive impairments of any cognitive function while maintaining independence [1]. The prevalence rate of MCI ranges from 6% in the population over 60 years of age [2] up to 25% for ages 80–84 [3]. Importantly, MCI is often considered a prodromal stage of dementia, especially considering that neuropathological changes of dementia may develop many years before the diagnosis, presenting both cognitive and behavioral symptoms previously the patients lose their independence [4]. The rates of progression of MCI due to Alzheimer’s disease (AD) to dementia have been estimated at between 8 and 15% [5, 6], which increases up to 80–90% after approximately 6 years [7–12]. Timely diagnosis of MCI is essential for identifying patients who are likely to progress to dementia and implementing early interventions to delay the pathological progression. Lifestyle interventions for MCI in its early stages may help to delay the onset of dementia [13]. Therefore, improving the assessment of MCI, including the incorporation of biomarkers in the usual clinical diagnostic procedures, could be critical for developing better diagnostic tools for MCI, particularly in determining its cause. Currently, much of the research on MCI has focused on MCI due to AD. Thus, incorporating biomarkers into clinical diagnosis procedures may help identify patients who are likely to develop AD and facilitate earlier intervention.

### Biomarkers in MCI

Biomarkers are objective measures that evaluate normal biological processes, pathological processes, or pharmacological responses to therapeutic interventions [14]. In the field of AD, neuroimaging and cerebrospinal fluid (CSF) protein analysis are the most widely used biomarkers [15]. Neuroimaging biomarkers include magnetic resonance imaging (MRI) to evaluate brain atrophy [16–18], positron emission tomography (PET) with F18-fluorodeoxyglucose (FDG) to measure glucose metabolism in different regions of the brain showing neuronal loss and neurodegeneration [19–22], and PET with tracers to detect amyloid-beta (Aβ) and Tau proteins in vivo [23–25]. CSF protein analysis of Aβ and Tau in their total and phosphorylated forms is also a validated biomarker for AD [26]. These biomarkers play a crucial role in the early and accurate diagnosis of AD, enabling earlier intervention and improving patient outcomes.

#### Fluids biomarkers

Peripheral biomarkers are of great interest because they are less invasive, less expensive, and more accessible. These types of biomarkers include molecules such as proteins, peptides, nucleic acids, microRNAs (miRNAs), lipids, and metabolites which can be detected in several biological fluids such as plasma, serum, urine, saliva, exosomes, or cellular components [27, 28]. Aβ proteins in their 42 amino acid form, which form amyloid plaques,total tau (T-Tau), which reflects the intensity of neurodegeneration; and phosphorylated tau (p-Tau), which correlates with the production of neurofibrillary tangles, are measured in CSF, and they are currently validated as diagnosis support in AD [15]. Despite the advances in biomarker research, no validated biomarkers are available for the diagnosis of MCI due to AD. Some studies showed lower concentrations of Aβ (Aβ1–40, Aβ1–42, and Aβ1–42/Aβ1–40 ratio) in CSF of MCI patients compared to healthy controls, reflecting higher brain Aβ concentrations and progressive cognitive impairment [29–34]. Furthermore, the detection of total and phosphorylated Tau (p-Tau) in CSF samples has also been used to diagnose MCI and AD with at least 85% sensitivity and 80% specificity, indicating neuronal damage and predicting progression from MCI to AD [35]. The combination of Aβ1–42 and Tau has demonstrated a high sensitivity of 95% and specificity of 83% in predicting the progression of MCI to AD [36].

Although blood levels of Aβ and tau have been evaluated as potential biomarkers for cognitive impairment, their concentrations are lower in blood compared to CSF, making their detection more challenging. Additionally, studies investigating Aβ42 and Tau levels in subjects with cognitive impairment have produced inconsistent results [37].

Recent research has focused on neurofilament light chain (NfL) as a potential biomarker for neurodegenerative diseases, including AD [38–43]. Studies have shown that plasma levels of NfL are significantly higher in patients with AD and MCI compared to controls [44] and are associated with cognitive and neuroimaging features [45–47]. Another potential blood biomarker is microRNAs (miRNAs), which play a role in regulating gene expression in the brain [48, 49]. However, validation studies are needed before miRNAs can be used clinically as a biomarker for MCI [50].

#### Genetics in MCI due to AD

Autosomal dominantly inherited forms of AD are present early in life; however, most early cases do not show a clear pattern of inheritance (2–10%); however, genetic predisposition to non-Mendelian inheritance of AD is high, with an estimated heritability of 80% [51]. Three genes including amyloid precursor protein (APP), presenilin 1 (PSEN1), and PSEN2 with fully penetrant mutations have been discovered as a cause of autosomal dominant AD, accounting for 5–10% of the occurrence of early AD. In addition to the above, the ε4 allele of the apolipoprotein E (ApoE) gene was identified as a strong risk factor for both early- and late-onset AD, where heterozygous carriers of the ε4 allele have an estimated threefold risk of developing AD and 15-fold risk in homozygous carriers of this allele [52–57]. In addition, at least 21 genetic risk loci have been identified in genome-wide association studies (GWAS) and mass sequencing that demonstrate how complex and multifactorial AD is in genetic terms [58, 59].

### Machine learning for medical diagnosis

Machine learning (ML) algorithms have been proposed as a promising tool to integrate multiple biomarkers for early detection, diagnosis, and prediction of dementia. ML models can analyze large amounts of data and identify complex patterns that may not be visible to human experts. Furthermore, ML algorithms can integrate data from different sources such as neuroimaging, genetics, and clinical data to develop models that can accurately predict the onset and progression of dementia [60–62]. Studies have shown that ML algorithms can improve the accuracy of dementia diagnosis and prediction compared to traditional methods based on single biomarkers [63].

ML can also help clinicians develop personalized treatment plans based on the individual patient’s biomarker profile and disease stage. By analyzing patterns in data from imaging, genetic, and biomarker assays, ML algorithms can identify the best treatment options and predict the effectiveness of specific interventions for individual patients. This can help improve the accuracy and effectiveness of treatment, potentially leading to better outcomes and improved quality of life for patients with Alzheimer’s disease and other forms of dementia [64].

In general terms, ML algorithms allow robust enquiries on many datasets to find patterns and relationships among the data [65]. There are some variations of how to define the types of ML algorithms but commonly they can be divided into categories according to their purpose and the main categories are the following: supervised learning, unsupervised learning, semi-supervised learning, and reinforcement learning [66]. Utilizing ML in large-scale data analysis, and taking into account the Four V’s of big data: volume, velocity, variety, and veracity [67, 68], is revolutionizing the production of scientific knowledge, by enabling novel and highly efficient ways of designing and evaluating research [69]. It is important to point out that this efficiency is given by the optimization of the use of resources to collect massive quality data in medical and healthcare contexts [70]. To the extent that the processes involved in the massive generation of scientific data are strategically optimized, as a consequence, ML analyzes and models acquire greater versatility and are potentially more scalable in their development and continuous improvement [71]. In that sense, one of the interests of developing ML research strategies using fluid biomarkers data is related to exploring ways of adjusting optimization gaps of cost-efficiency in the use of resources to improve the diagnosis from MCI to AD on a large scale.

Without a doubt, ML offers interesting new data research opportunities. However, in the medical community, there is great concern about the development of medical applications based on ML models [72, 73]. This is because as ML algorithms become more advanced, it is more challenging to comprehend and retrace how the algorithm came to a result, which translates into a trust issue due to the lack of explainability that these models have [74]. The whole calculation process used by an ML algorithm is turned into what is commonly referred to as a “black box” that is impossible to interpret. These black box models are created directly from the data, and not even the researchers who create the algorithm can understand or explain what exactly is happening inside them or how the ML algorithm arrived at a specific result [75]. Many of the ML algorithms cannot explain how and why they have issued a given answer or decision [76]. This occurs mainly in modeling approaches based on neural networks (one of the most popular in use) [77]. In the given context, explainable artificial intelligence (XAI) is a rapidly growing research area within the realm of machine learning. It focuses on uncovering the ways in which these algorithms make decisions that are considered “black box,” by examining the measurements and rules at play and assisting in making the modeling process more self-explanatory. In that sense, the XAI becomes increasingly crucial for machine learning-driven applications, especially in medical diagnosis [78]. Essentially, for the successful development of machine learning-based applications to improve the diagnosis from MCI to AD either from fluid biomarkers or multimodal data, it is necessary that the explainability of the model be clear and consistent from all possible perspectives, in coherence with its theoretical and experimental framework [79].

To fully realize the potential of ML in this context, the purpose of this research is to conduct a systematic review of existing studies on the use of machine learning and fluid biomarkers in dementia research. This review aims to identify gaps in our understanding of the relationships between different biomarkers, highlight areas where additional research is needed, and provide guidance for the design of future studies in this field. The combination of machine learning and fluid biomarkers research holds enormous promise for advancing our understanding of dementia pathobiology, and a systematic review of the existing literature is a crucial step towards realizing this potential.

## Materials and methods

For this research, we followed the Preferred Reporting Items for Systematic Reviews and Meta-Analyses (PRISMA) checklist and statement.

### Identification of studies

We searched PubMed Central and Scopus databases. PubMed Central is the digital archive of the United States National Institutes of Health which was selected for its wide scope and its relevance in the biomedical and life sciences. Scopus is a wide database containing peer-reviewed abstracts and citations. We performed a sensitive literature search by using specific keywords that were defined in four categories (including synonyms and related words) that allow to maintain an accurate search: (1) MCI—mild cognitive impairment and MCI; (2) Diagnosis—diagnosis and diagnostic; (3) Machine learning—machine learning, artificial intelligence, algorithm, and deep learning; and (4) Fluid biomarkers—Tau, cerebrospinal fluid, amyloid-beta, fluid biomarker, miRNA, microRNA, blood, serum, plasma, urine, saliva, progranulin, and neurofilament. The search queries were carried out defining that at least one of the keywords of each category considered appeared in the title, abstract, or keywords of the article. We focused on articles published between January 1, 2012, and January 1, 2023, to base our analysis on recent studies.

### Selection of studies

**Fig. 1:**
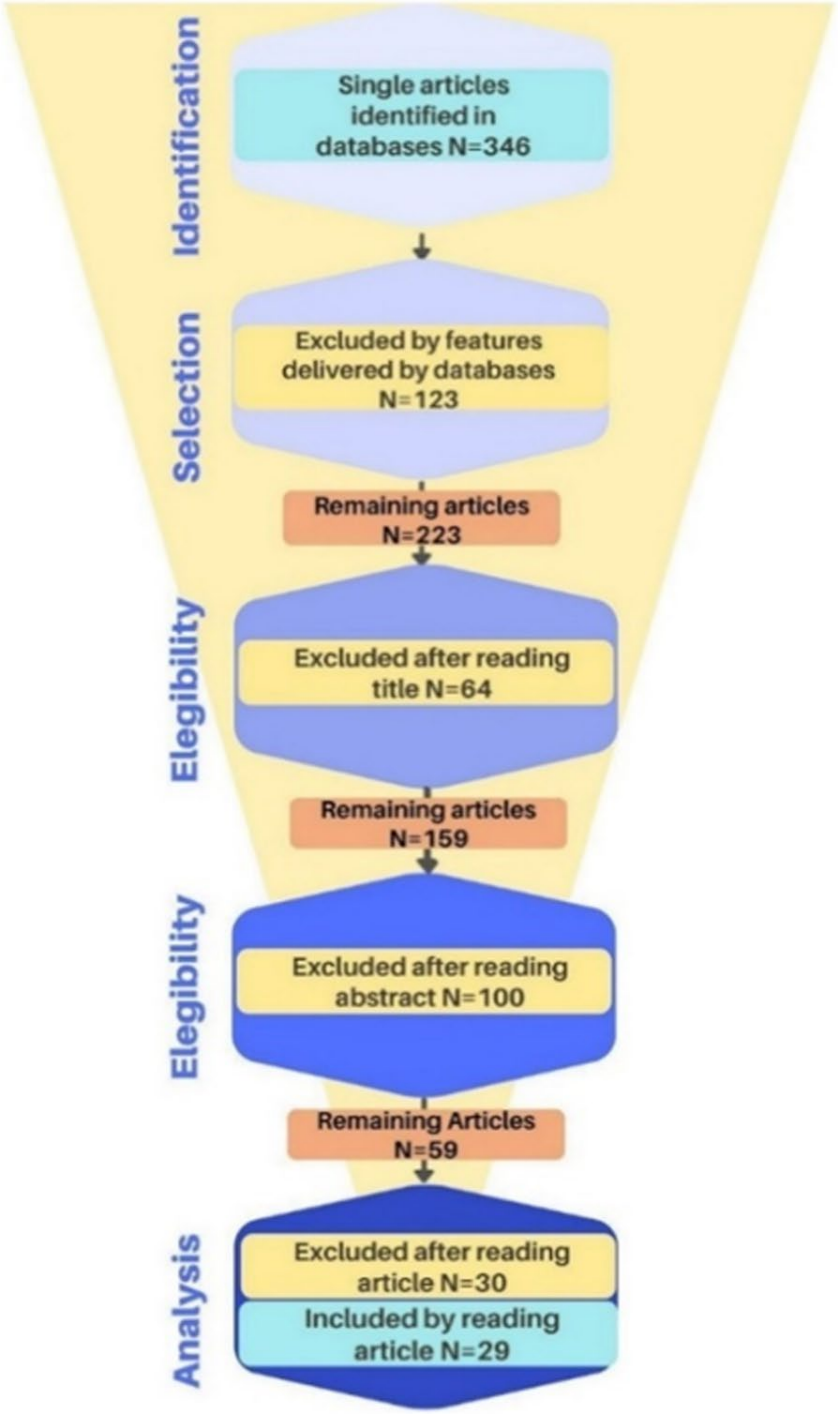
Systematic literature search flow diagram (PRISMA). This diagram starts with the total number of records identified through database searching. From there, the diagram outlines the number of records screened. Then, it indicates the number of records excluded after the initial screening, typically because the titles or abstracts clearly indicate that the studies do not meet the inclusion criteria. Next, the diagram shows the number of full-text articles assessed for eligibility, followed by the number of full-text articles excluded and the reasons for their exclusion. Finally, the diagram presents the number of studies included. This process makes the selection process transparent, which is crucial for the credibility of the systematic review

All abstracts were screened by co-authors. The full text of selected abstracts was assessed for eligibility by co-authors, and conflicts were resolved by consensus. Firstly, a search was carried out for the selected keywords using Boolean operators in the two aforementioned databases. Secondly, the articles were checked for the inclusion and exclusion criteria by features found in the databases. After that, we read the titles of all the remaining articles to check if the articles were within the scope of our study and considered at least one biomarker other than neuroimages (fluid, genetic, or clinical data). In the same way, we proceeded to read the abstracts of the remaining articles. Finally, after reading these, we selected the articles that were included in this review. The flowchart in Fig. 1 shows the sequence of actions and the outcomes.

### Inclusion and exclusion criteria

Studies were eligible if (i) the article described empirical, quantitative, longitudinal studies; follow-up studies; neuroimaging studies; randomized controlled trials; quasi-randomized controlled trials; and cross-sectional studies, based on human populations all over the world, and (ii) considering samples of mild cognitive impairment in conjunction with machine learning and fluid biomarkers, in which the algorithm has validation.

Studies were excluded if they were (i) review articles, conference abstracts, and studies without a complete set of data or with no algorithm validation.

### Categorization of studies

Specific data from each study was recorded on a table including all the relevant citation information: digital object identifier (DOI), authors, title, and year of publication. Then, the important information in each paper was included: abstract, size, and origin of the cohort, which machine learning algorithms were used, performance of the best algorithm, features, number of features, and validation technique. Thirdly, after reviewing each paper, it was classified by the type of feature. In that sense, all the papers found in this systematic review fall into the category of supervised ML algorithms.

Supervised learning is the most common ML approach in which an algorithm learns to make predictions or decisions based on labeled input–output pairs [80]. In this approach, the learning model is provided with training data, which consists of input features and corresponding output labels. The goal of the algorithm is to learn the relationship between the input features and the output labels, which can then be used to make predictions on new, unseen data. Also, it is important to explain that supervised machine learning methods can be for classification and/or regression outputs. Classification deals with the task of predicting discrete output labels or categories, whereas regression involves predicting continuous output values. Classification models are evaluated using metrics such as accuracy, precision, recall, and F1-score, while regression models are evaluated using metrics like mean squared error (MSE), root mean squared error (RMSE), mean absolute error (MAE), and *R*-squared. The main algorithms for classification are as follows: (1) logistic regression—a linear model for classification that uses the logistic function to estimate probabilities [81], (2) decision trees—a tree-like structure that recursively splits the input space based on feature values to make predictions [82], (3) support vector machines—a method that finds the optimal hyperplane to separate the different classes [83], (4) random forest—an ensemble learning method that constructs multiple decision trees and combines their output [84], and (5) neural networks—a method to make predictions by being trained on a labeled dataset, where input–output pairs are provided. One of the most popular supervised learning algorithms for neural networks is backpropagation [85]. On the other hand, the main algorithms for regression are as follows: (1) linear regression—a linear model that predicts the target variable by minimizing the sum of squared errors [86], (2) Lasso regression—a linear model that includes L1 regularization, which helps in feature selection and reducing overfitting [87], (3) ridge regression—a linear model that includes L2 regularization, which helps in reducing overfitting [88], (4) decision trees (for regression)—similar to classification trees but predicting continuous values instead of classes [82], (5) neural networks (for regression)—similar to classification neural networks but optimized for continuous output predictions [89].

**Fig. 2:**
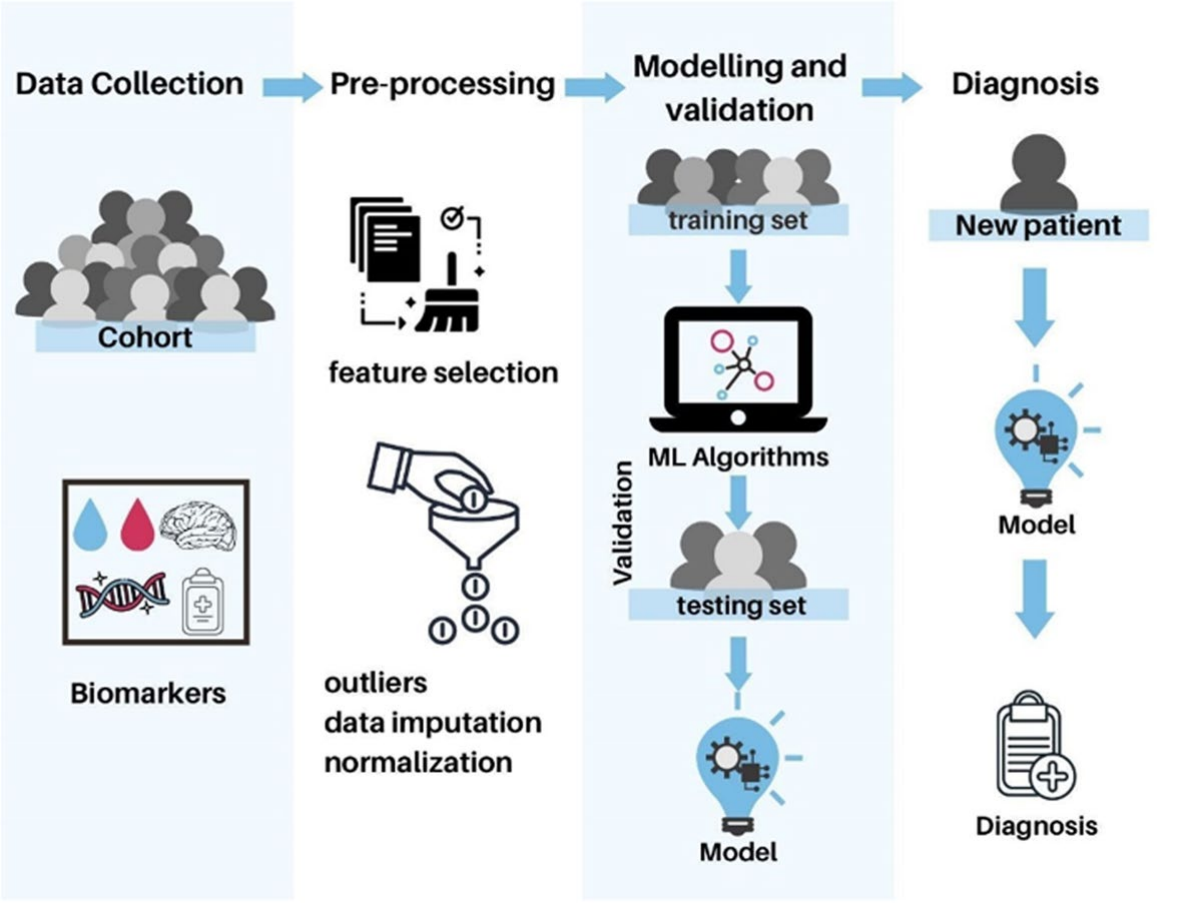
Supervised machine learning (ML) process. The first step is when biomarkers are taken from a cohort, then the data is pre-processed, outliers are removed, data is imputed and normalized, then the data is divided between testing and training data sets; the first is used to train the algorithms, and the second test it and validate it, if the model is selected as the best performing algorithm. Finally, the model can be used to diagnose a new patient

In summary, according to the above definitions, all reviewed articles can be categorized for classification purposes based on supervised ML algorithms (Table 1). The diagram of Fig. 2 represents a supervised learning process for medical diagnosis using biomarkers.

**Table 1:**
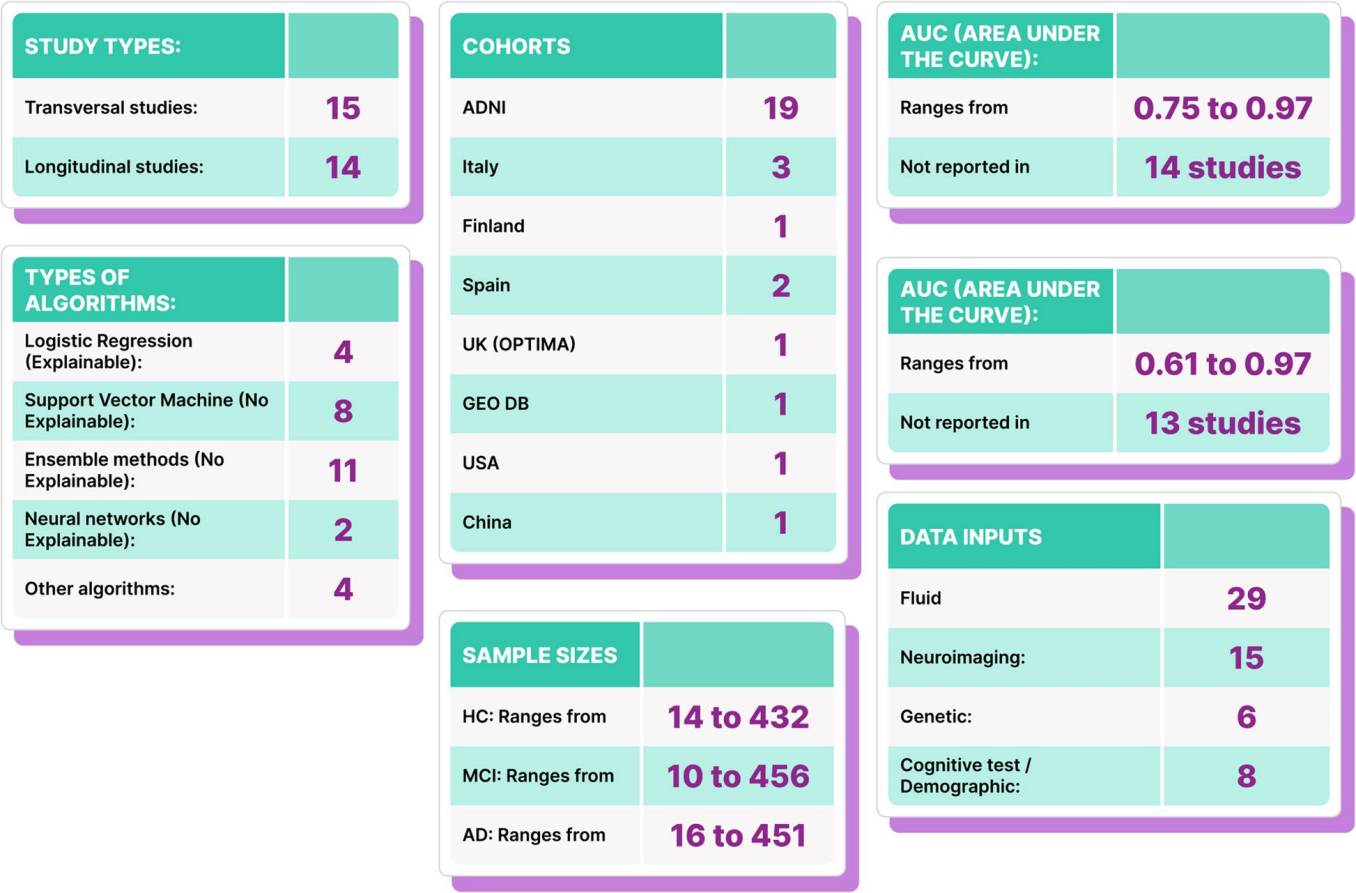
Review’s descriptive summary

## Results

Our search identified 346 articles published between 1/2012 and 1/2023, of which 123 studies were excluded based on features delivered by databases. Sixty-four studies were excluded after reading the title. One-hundred studies were excluded after reading the abstract. Thirty studies were excluded during full-text screening. Finally, 29 studies meet our criteria for this review of MCI using machine learning as a diagnostic tool.

The systematic review highlights the variety of machine learning algorithms used in diagnosing MCI and AD, with traditional methods being more common in transversal studies and a diverse set of other algorithms used in longitudinal studies. Traditional machine learning methods, such as support vector machine and random forest, are the most common, but other algorithms like logistic regression methods and non-traditional techniques like extreme learning machine are also present. The Alzheimer’s Disease Neuroimaging Initiative (ADNI) is the most common cohort source. Sample sizes for the groups HC, MCI, and AD vary significantly across studies. AUC and ACC values also vary across studies, but not all studies report both values. Some of the highest AUC values are found in Redolfi et al. [90] and Sh et al. [91], while high ACC values are reported in Khatri et al. [92] and Barbará-Morales et al. [93]. Table 1 represents a descriptive summary of the review.

### Study types

We identified 2 main groups among the selected papers, those that use cross-sectional data and those that use longitudinal data from the established cohort (Table 1). Almost half of the selected articles correspond to each of the categories mentioned (15 transversal and 14 longitudinal). Also, we found that the Alzheimer’s Disease Neuroimaging Initiative (ADNI) is the widely used cohort for these studies. Another cohort is the Oxford Project to Investigate Memory and Aging (OPTIMA), while the rest are their own non-public cohorts (Table 2).

**Table 2:**
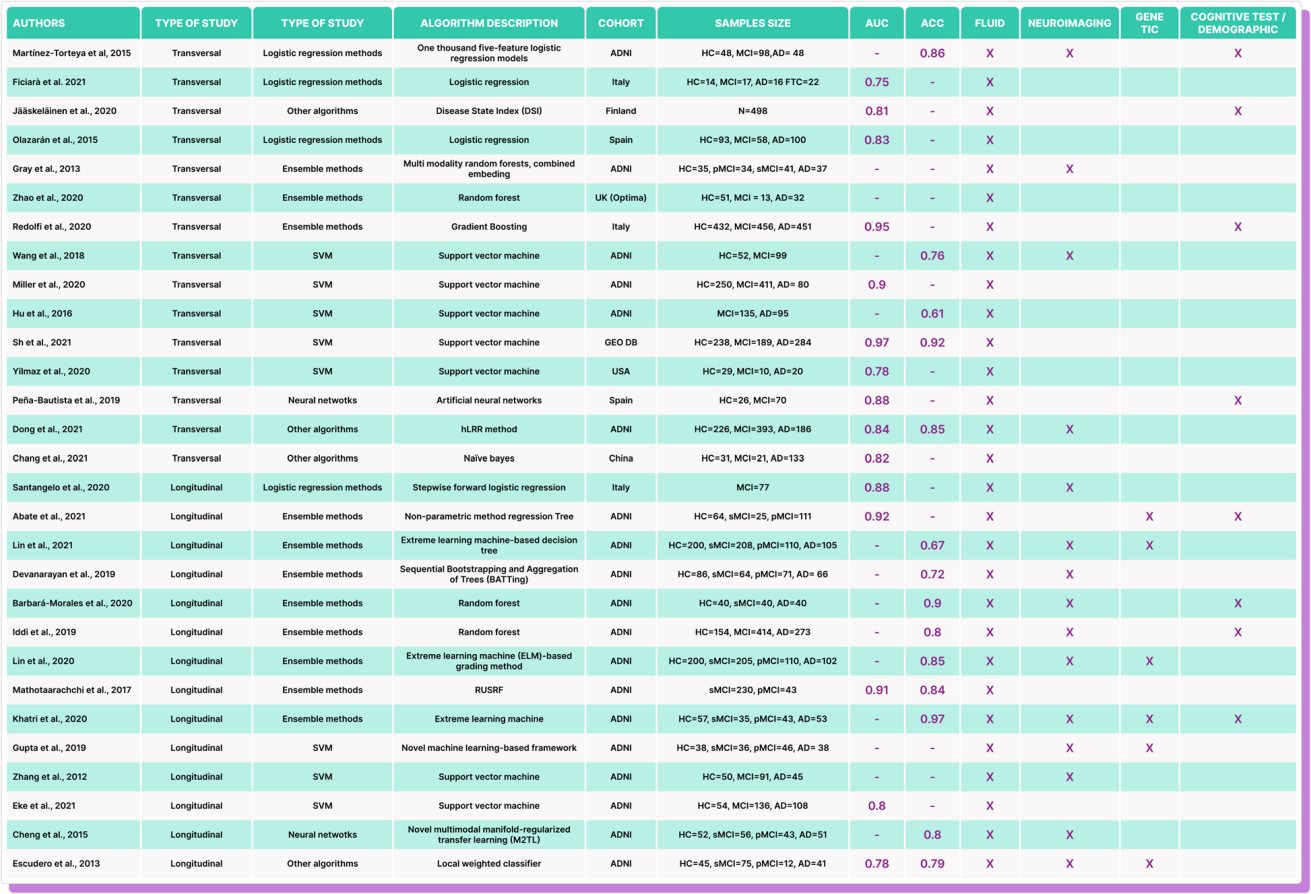
Comprehensive synthesis of the systematic review findings

References: Martínez-Torteya et al, 2015 [94], Ficiarà et al. 2021 [95], Jääskeläinen et al., 2020 [96], Olazarán et al., 2015 [97], Gray et al., 2013 [98],  Zhao et al., 2020 [99], Redolfi et al., 2020 [90], Wang et al., 2018 [100], Miller et al., 2020 [101], Hu et al., 2016 [102], Sh et al., 2021 [91],  Yilmaz et al., 2020 [103], Peña-Bautista et al., 2019 [104], Dong et al., 2021 [105], Chang et al., 2021 [106], Santangelo et al., 2020 [107],  Abate et al., 2021 [108], Lin et al., 2021 [109], Devanarayan et al., 2019 [110], Barbará-Morales et al., 2020 [93],  Iddi et al., 20119 [111], Lin et al., 2020 [112], Mathotaarachchi et al., 2017 [113], Khatri et al., 2020 [92], Gupta et al., 2019 [114], Zhang et al., 2012 [115], Eke et al., 2021 [116], Cheng et al., 2015 [117], Escudero et al., 2013 [118]

Transversal studies demonstrate a higher number of algorithms focused on logistic regression methods and traditional machine learning methods. The highest reported ACC is 0.86 [94]. Longitudinal studies utilize a more diverse set of algorithms, including other algorithms such as extreme learning machine and novel machine learning-based frameworks. The highest reported ACC is 0.97 [92].

### Algorithms inputs

Regarding the characteristics used as input for the algorithms (Table 1), 10 articles were found to use only fluid biomarkers as input for the algorithm, and the others use it accompanied by other types of biomarkers, specifically 15 use neuroimaging, 8 neuropsychological tests, and 6 genetic information in addition to fluid biomarkers (showing that the tendency is to employ more than one source of information, in a multimodal way).

In fact, we observed that longitudinal studies tend to be multimodal, applying different types of inputs. Importantly, we found that genetic data is the least used feature in ML algorithms while the most used is neuroimaging data, considering that this study only included articles that used neuroimaging in support of fluid biomarkers. However, only 14 articles did not use neuroimaging as input to the algorithm, corresponding to less than 50% of the included studies. Also, we found that algorithms using only fluid biomarkers as features have reported very good performances.

### Cohorts

Regarding patients’ home country, 28 cohorts include patients from the USA, 4 from Italy, 3 from Spain, and 2 from Korea, and with one study each, considering patients from China, Holland, Finland, and the UK were found (Table 1). In addition, we identified that most of the included studies consider three main classification categories as targets for their patients: healthy control (HC), MCI, and AD. However, we also found three studies that considered only HC and MCI, one that considered MCI and AD and 2 that considered only MCI divided into two subcategories (progressive and stable) (Table 1).

Studies using the ADNI cohort have a broad range of sample sizes and algorithm types. Performance metrics (AUC and ACC) also vary considerably within this group. Studies from Italy, Spain, and other countries generally report high AUC and ACC values, though sample sizes are often smaller than those in ADNI cohort studies.

### Performance metrics (AUC and ACC)

**Fig. 3:**
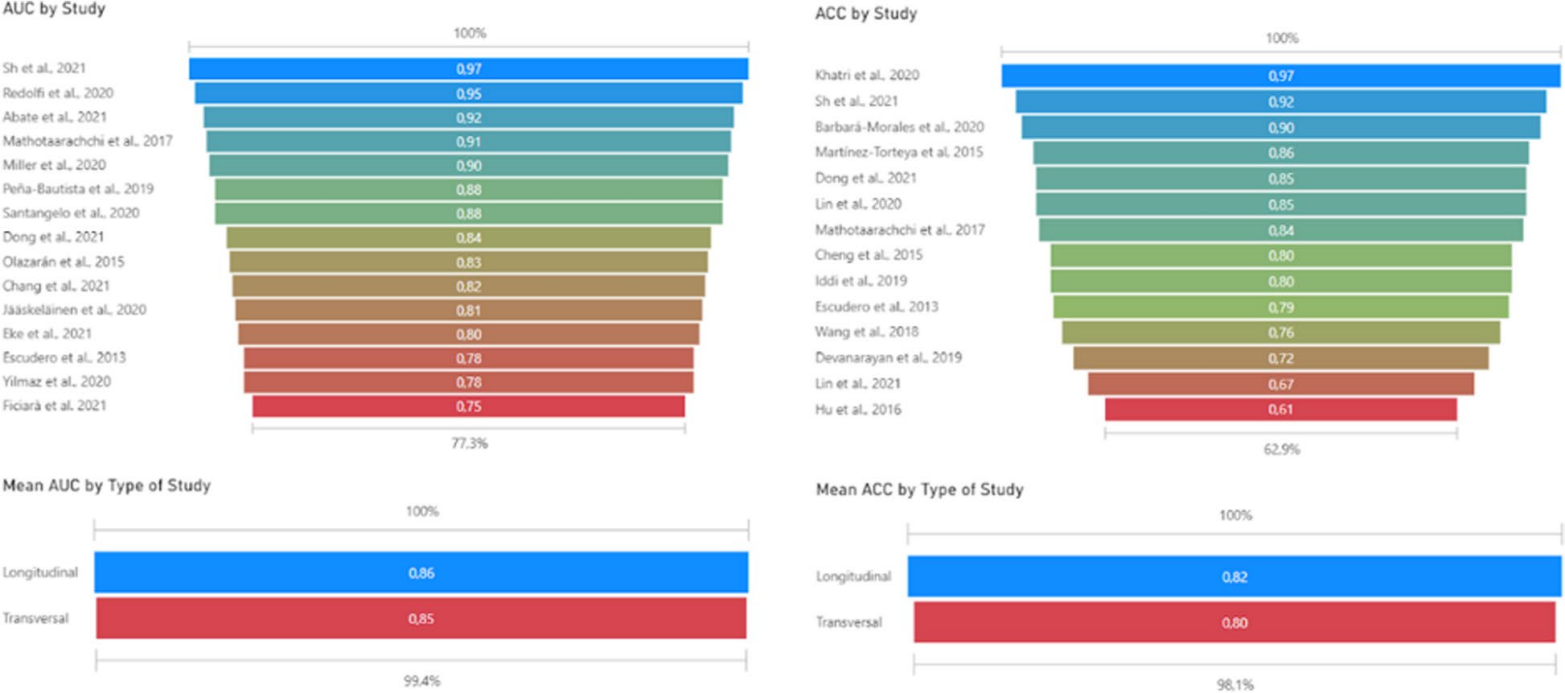
Funnel plot of algorithm performance by study. This funnel plot is a specialized form of the scatterplot, uniquely tailored for the analysis and visualization of data behavior between minimal and maximum metrics. Its primary function is to assist in identifying anomalies or outliers within the data set. In a funnel plot, data points are depicted as dots and plotted within a funnel-shaped graphical field. The shape of the funnel serves as a visual guideline, delineating the expected range of variation based on statistical norms. Consequently, any data point, or dot, that is plotted outside this funnel shape is classified as an outlier, indicating a substantial deviation from the anticipated pattern or range. In the context of this review, it is noteworthy that all the metrics derived from the studies are plotted within the confines of the funnel. This suggests that there is a consistent pattern in the data, with no significant anomalies or outliers detected. It implies that the metrics of the studies fall within the expected range and adhere to the statistical norms, reinforcing the reliability and validity of the reviewed studies

In machine learning, area under the curve (AUC ROC) and accuracy (ACC) are two distinct performance metrics used to evaluate the effectiveness of classification models. AUC refers to the area under the ROC curve, which plots the true-positive rate (sensitivity) against the false-positive rate (1-specificity) at various threshold settings. AUC ranges from 0 to 1, with a higher value indicating better model performance. It is particularly useful when dealing with imbalanced datasets, as it considers both sensitivity and specificity. ACC, on the other hand, is the ratio of correct predictions to the total number of predictions made. It measures the overall performance of a model and is more suitable for balanced datasets. However, ACC can be misleading in the case of imbalanced datasets, as it may not account for the true effectiveness of a classifier. In summary, AUC is a more robust performance metric that considers both sensitivity and specificity, while ACC measures the overall performance but may be less informative for imbalanced datasets [119]. In this review, AUC values are generally high, with most reported values above 0.8. The highest AUC value is 0.97 [91], achieved using a support vector machine in a transversal study. ACC values also tend to be high, with most reported values above 0.8. The highest ACC value is 0.97 [92], achieved using an extreme learning machine in a longitudinal study (Fig. 3). In some cases, only one of the two performance metrics is reported, making it difficult to comprehensively compare the algorithms’ performances. No significant difference is observed between the accuracy achieved between the two groups, nor in the reported AUC. However, longitudinal studies have the potential of predicting the diagnosis of diseases, which could allow early action to use treatments aiming to delay the progression of the disease.

**Fig. 4:**
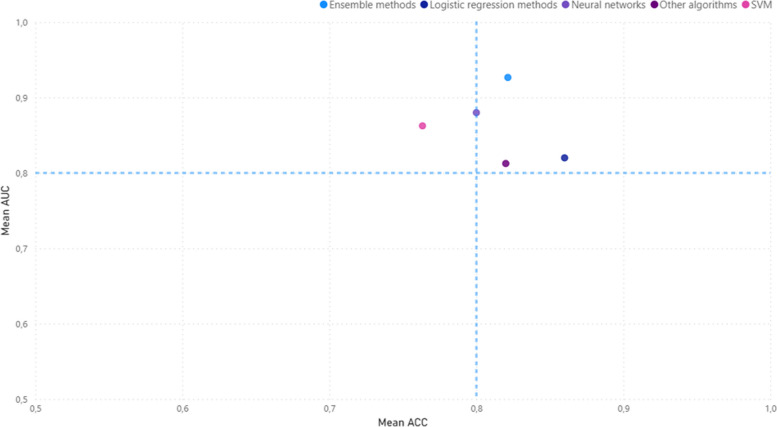
Scatterplot of algorithm performance. Scatterplot representing the performance of the ML algorithms. The *x*-axis, labeled “ACC,” measures the accuracy of the algorithms. Accuracy is a simple metric for binary classification problems, representing the proportion of true results (both true positives and true negatives) among the total number of cases examined. The *y*-axis, labeled “AUC,” represents the area under the receiver operating characteristic (ROC) curve. AUC is a popular metric in machine learning for binary classification problems. It measures the tradeoff between a true-positive rate and a false-positive rate. An AUC of 1.0 means the model has a perfect classification, while an AUC of 0.5 implies the model is no better than random guessing. Each point in the scatterplot represents a different machine learning algorithm. The position of the point on the graph shows the performance of the algorithm on both metrics: its accuracy and its AUC score. The scatterplot also features a performance target of 0.8. This could be represented as a line or a highlighted area in the plot, indicating the desired minimum performance level for both the accuracy and AUC. Algorithms that fall within or above this target region are considered to meet or exceed the performance goal. This visual comparison makes it easier to quickly identify which algorithms meet the performance target according to these two key metrics

Overall, logistic regression methods generally report high ACC values (0.86 and 0.88), though AUC values are not always available. Traditional ML methods, specifically Support Vector Machines, are widely used in transversal studies with varying ACC values (0.61 to 0.97). Ensemble methods (i.e.: Random Forest algorithms) are more common in longitudinal studies and report ACC values between 0.8 and 0.9. Other algorithms show a wider range of performance, with AUC values from 0.75 to 0.97 and ACC values from 0.67 to 0.97 (Fig. 4).

### Black-box algorithms and sample sizes

In relation to the black-box problem mentioned, of the total of 29 studies reviewed, 11 (38%) of them use algorithms of neural networks, support vector machines, and random forests, which are considered within the black-box methods [120]. Importantly, relative to the sample sizes of the studies included here (Table 1), it is observed that of a total of 18 studies (62%), the sample is less than 100 cases. Of the 15 cross-sectional studies, 8 (53%) of them have a sample size of less than 100. In longitudinal studies, a total of 10 (71%) use a sample of less than 100 cases. A very relevant aspect of modeling based on ML is related to the sampling size and the power of its estimates. Using ML on small-size datasets could present a problem. The smaller the dataset, the less powerful and less accurate the models [121]. The process of ML involves training, validation, and test datasets [122]. This perspective is essential in the development of models and must be considered.

For the cross-sectional studies, the most used types of techniques are the explicable ones with 4 articles and support vector machine (SVM) with 5 articles out of a total of 15, while for the longitudinal studies, the assemblies are the ones that take the lead with 7 out of 14 articles. The category of explainable models includes logistic regression and decision tree.

### Methodological issues

No article was found in this review that considered the longitudinal dimension within the algorithm itself. However, in all cases, this dimension is delivered to the data label with which the algorithm will be trained later. For the use of longitudinal data, deep learning methods are the most recommended in the literature but considering that these fields the availability of data is limited, and it is also sought that the methodology used be interpretable, which excludes efforts with deep learning that have an advantage in the representation of longitudinal data [123]. The classification targets of the algorithm are relevant for the comparison of their results. It is possible to differentiate between classification targets which include disease progression, which require a longitudinal study to be able to evaluate the individuals at least in two different time points, and classification targets without progression, where the targets are defined on a single data point. In the case of classification targets that include disease progression, they are usually built to represent the longitudinal dimension of the data. For example, in a study, 4 classes were used (healthy control (HC), cognitive impairment without progression to Alzheimer’s (sMCI), cognitive impairment with progression to Alzheimer’s (cMCI), and Alzheimer’s (AD)).

## Discussion

The growing use of artificial intelligence techniques today to work new diagnostic algorithms with current data has an impact on the diagnostic tool, improving its accuracy and helping to predict the status or possible evolution of the patient. Biomarkers can play a very important role [124, 125] which could distinguish between AD and MCI or between MCI and age-related changes [126]. There are different types of biomarkers, some of which are better studied, such as those obtained from cerebrospinal fluid and neuroimaging, and novel types, especially due to their cost-efficiency, such as fluid biomarkers that include those obtained from the blood, urine, and saliva, which correspond to proteins and miRNA, among others [127–129]. On the one hand, they would allow greater access to the population and, on the other, to the investigation of their use for diagnosis, increasing sample sizes, especially when we talk about ML tools, where the sample size becomes relevant in order to achieve greater statistical power.

In this review, it is seen that the sample sizes tend to be low; however, the article by Redolfi et al. stands out with 1339 participants between HC, MCI, and AD for the articles of the cross-sectional category, using the base built from of a Medical Informatics Platform installed across 3 Italian memory clinics and the article by Iddi et al., with 841 participants between controls, MCI, and AD for the longitudinal articles using the ADNI database. Then, articles with a sample size of up to 59 patients were included [103], where an AUC of 78% was obtained, in this case, the MCI category has 10 participants, while HC and AD have 29 and 20 patients, respectively, indicating a very low statistical power.

Today the diagnosis of MCI is based mainly on neuropsychological tests that include cognitive and functional tests. These tests can be influenced by various factors such as age, education, and lifestyle, among others [130, 131], leading to a focus on new forms of detection that are more reliable and ideally easily accessible. Neuropsychological tests can provide complementary information to suggest the etiological diagnosis, but not enough to do it on their own. Here, we carry out a systematic review of studies from the last 10 years that consider ML techniques and use fluid biomarkers for the diagnosis of MCI due to AD. As for the selected articles, most use some type of biomarker in addition to fluid biomarkers, as complementary information. It was found that fluid biomarkers are mainly added to neuroimaging characteristics, neuropsychological tests, and genetic information. Being neuroimaging is the most used, especially in longitudinal articles, this may be due to the amount of neuroimaging data available to test new ML architectures in databases such as ADNI, compared to the datasets of other biomarkers, which facilitates the implementation and investigation of these techniques.

Another point to highlight is the explainability of the models to be used, in terms of biomedicine and diagnostics in general; it is expected to be closer to white-box than black-box type models, since an important component is the process of the architecture of the model. In this case, there are 4 explainable models for cross-sectional articles, corresponding to different logistic regressions, while for longitudinal articles, there are only two, a logistic regression and a decision tree. While the other models lose explainability to a certain degree, reaching black boxes such as the neural network that only appears in 2 articles in this review [132, 133] where the explainability of the process is completely lost.

In addition, a relevant aspect in the development of diagnostic models for MCI is related to the cultural representativeness gap [134]. There is no single cause of AD, but multiple factors are involved [135]. Among these factors, the socioeconomic and cultural condition is very important for both diagnosis and treatment [136, 137]. Unfortunately, most research in MCI and AD is conducted in the US and European population, where findings and inferences cannot be extrapolated cross-culturally to everyone in appropriate cultural contexts and niches [138–143]. This premise is applied in this specific review of the diagnosis of MCI with the use of ML, where there is no article on the Latin American or African population and the majority includes the population of the USA and/or Europe, leaving a gap in the study of these related populations.

On the other hand, it is important to highlight that most of the ML approaches mentioned in this review are based on associative inference. Primarily, these methods propose a diagnostic framework that establishes correlations among various factors including symptoms, neuroimaging data, neuropsychological tests, genetic information, and other relevant variables. However, associative inference is the simplest in a hierarchy of possible inference schemes because it does not allow causal explanations to be attributed to the data [144]. Instead, an approach based on causal and/or counterfactual inference modeling would allow it [145]. There is evidence that a highly accurate medical diagnosis can be performed based as a counterfactual inference approach using ML methods such as probabilistic graphical model (PGM), Bayesian networks, and noisy-OR algorithms [146]. Under this approach, it is argued that diagnosis is the process of finding causal explanations for a patient’s symptoms. This implies promoting causal reasoning to show that the probability of occurrence of an effect B has really been caused by cause A. This leads to developing a diagnostic measure to classify the probability that a disease X is causing the symptoms of a patient given the evidence. In general, the criticism leveled at associative inference is the fact that not separation of correlation from causation places strong constraints on the accuracy of associative diagnostic algorithms, sometimes leading to suboptimal diagnostic results [147]. In this case, these methodological perspectives of causal inference would be very helpful for the development of MCI and AD diagnostic models based on ML algorithms.

## Conclusions and future directions

The latest advances in neuroimaging, laboratory analysis, genetic, and ML techniques have led to a progressive change in the diagnosis of neurodegenerative diseases. Overall, it is not possible to determine with our systematic review a group of techniques or features which achieves better results than others, since the metrics reported vary widely. However, it is possible to see that the following points address the major themes and challenges that appear in the article and would be essential considerations for any researcher planning to embark on a similar research journey in the realm of neurodegenerative diseases using ML techniques:

- Multi-modal approach: The growing trend towards a multi-modal approach in utilizing neuroimaging, laboratory, genetic, and ML techniques from cohorts like the Alzheimer’s Disease Neuroimaging Initiative, Neuroimaging in Frontotemporal Dementia, and UNITED Consortium [63] requires careful planning and integration. Researchers need to understand how to synergize various data types and technologies, leveraging them for more accurate diagnoses.
- Longitudinal data challenges: The lack of sufficient data, short monitoring time, and a need for interpretable results in longitudinal studies present significant challenges. When planning research, it is essential to ensure that the methodology allows for long-term monitoring and that the tools used can handle sparse or incomplete data.
- Cultural representativeness and diverse population sampling: The absence of studies in underrepresented populations like Latin American, Caribbean, and African regions necessitates planning for more inclusive research [137, 142, 148, 149]. This could involve considering different cultural, socioeconomic, and demographic factors that may influence the disease progression and diagnosis.
- Use of white-box models: The emphasis on using white-box type machine learning algorithms reflects a need for transparency and interpretability in the models. Researchers need to carefully choose or design algorithms that not only perform well but also provide insights into how and why they are making specific predictions.
- Cost-effective large-scale studies: Achieving long-term multimodal longitudinal monitoring in a cost-effective manner is crucial. Planning must include budget considerations, the incorporation of large-scale sample sizes, and a strategic approach to collect and analyze the large quantities of data required.

In summary, the intricate and varied array of techniques within neuroimaging, laboratory analysis, genetics, and machine learning alludes to a captivating yet demanding trajectory in neurodegenerative disease research. Successful amalgamation of these methodologies demands thorough planning, inclusiveness, and transparency, thereby establishing the foundation for a groundbreaking era in diagnosing and comprehending these ailments. Furthermore, the establishment of a multidisciplinary task force is imperative to rectify diagnostic accuracy.

## Data Availability

All data generated or analyzed during this study are included in this published article.

## Abbreviations

MCI: Mild cognitive impairment
AD: Alzheimer’s disease
PRISMA: Preferred Reporting Items for Systematic Reviews and Meta-Analysis
CSF: Cerebrospinal fluid
PET: Positron emission tomography
FDG: F18-fluorodeoxyglucose
NFL: Neurofilament light chain
ML: Machine learning
